# Bioactive Thymosin Alpha-1 Does Not Influence F508del-CFTR Maturation and Activity

**DOI:** 10.1038/s41598-019-46639-1

**Published:** 2019-07-16

**Authors:** Andrea Armirotti, Valeria Tomati, Elizabeth Matthes, Guido Veit, Deborah M. Cholon, Puay-Wah Phuan, Clarissa Braccia, Daniela Guidone, Martina Gentzsch, Gergely L. Lukacs, Alan S. Verkman, Luis J. V. Galietta, John W. Hanrahan, Nicoletta Pedemonte

**Affiliations:** 1 Analytical Chemistry and In-vivo Pharmacology Facility, Fondazione Istituto Italiano di Tecnologia, via Morego 30, 16163 Genova, Italy; 2 UOC Genetica Medica, IRCCS Istituto Giannina Gaslini, Via Gerolamo Gaslini 5, 16147 Genova, Italy; 3 Department of Physiology, McGill University, Montréal, Québec Canada; 4 Department of Biochemistry, McGill University, Montréal, Québec Canada; 5 Marsico Lung Institute and Cystic Fibrosis Research Center, The University of North Carolina, Chapel Hill, NC USA; 6 Department of Medicine, UCSF, San Francisco, California, USA; 7 D3-Pharmachemistry, Fondazione Istituto Italiano di Tecnologia, via Morego 30, 16163 Genova, Italy; 8 Telethon Institute of Genetics and Medicine (TIGEM), Pozzuoli, Italy; 9 Department of Cell Biology and Physiology, The University of North Carolina, Chapel Hill, NC USA; 10 Department of Physiology, UCSF, San Francisco, California, USA

**Keywords:** Mutation, Chloride channels

## Abstract

Deletion of phenylalanine 508 (F508del) in the cystic fibrosis transmembrane conductance regulator (CFTR) anion channel is the most frequent mutation causing cystic fibrosis (CF). F508del-CFTR is misfolded and prematurely degraded. Recently thymosin a-1 (Tα-1) was proposed as a single molecule-based therapy for CF, improving both F508del-CFTR maturation and function by restoring defective autophagy. However, three independent laboratories failed to reproduce these results. Lack of reproducibility has been ascribed by the authors of the original paper to the use of DMSO and to improper handling. Here, we address these potential issues by demonstrating that Tα-1 changes induced by DMSO are fully reversible and that Tα-1 peptides prepared from different stock solutions have equivalent biological activity. Considering the negative results here reported, six independent laboratories failed to demonstrate F508del-CFTR correction by Tα-1. This study also calls into question the autophagy modulator cysteamine, since no rescue of mutant CFTR function was detected following treatment with cysteamine, while deleterious effects were observed when bronchial epithelia were exposed to cysteamine plus the antioxidant food supplement EGCG. Although these studies do not exclude the possibility of beneficial immunomodulatory effects of thymosin α-1, they do not support its utility as a corrector of F508del-CFTR.

## Introduction

Loss-of-function mutations occurring in the gene encoding the cystic fibrosis transmembrane conductance regulator (CFTR) protein cause cystic fibrosis (CF), the most frequent lethal genetic disease in Caucasian populations^1,2^. CFTR functions as a chloride channel expressed at the apical side of epithelial cells and the disease affects the lungs, pancreas, liver, exocrine glands and other organs. More than 2000 mutations have been described in the *cftr* gene, however the deletion of phenylalanine at position 508 (F508del) is the most frequent. F508del-CFTR displays several molecular defects including aberrant folding that results in premature degradation by the proteasome^3^, and if trafficked to the plasma membrane, reduced stability due to peripheral protein quality control mechanisms^4,5^ and low open probability (reviewed by^6^).

In the last 15 years efforts have been focused on the identification of small molecules that are able to restore defective processing (correctors) and activity (potentiators) of mutant F508del-CFTR^7–13^. VX-809^14^ or Lumacaftor™ (Vertex Pharmaceuticals Inc) was the first corrector drug to be approved by the FDA and is combined with the potentiator Ivacaftor (VX-770) in the drug Orkambi™ (Vertex Pharmaceuticals Inc) to treat CF patients homozygous for the F508del mutation^15^. VX-809 is a pharmacological chaperone that binds directly to F508del-CFTR and promotes its folding^16–18^. VX-809 binds to the helical subdomain of the first nucleotide binding domain (NBD1) at non-physiological concentrations^19^, however the presence of amino acids 370–380 in TM6 and the linker to NBD1 are necessary for the pro-folding action of VX-809^17,20^.

Combining correctors that have different mechanisms of action can improve the rescue of mutant CFTR^18,20–22^ through binding to multiple sites^20,22^ and/or through modulation of proteostasis, i.e. by altering cellular protein homeostasis so that CFTR processing and plasma membrane stability is improved^23–25^. Indeed, rescue of mutant CFTR trafficking has already been demonstrated through modulation of gp78, CHIP, CAL, RNF5/RMA1, Dab2, RPL12, KIFC1, RFFL and cCBL^5,25–35^. Autophagy is reportedly defective in CF cells and its restoration has been proposed as a therapeutic strategy to rescue mutant CFTR trafficking and function^36,37^. One compound proposed to correct F508del-CFTR maturation and functional expression through enhanced autophagy is cysteamine^37^.

Recently, it has been reported that the naturally occurring polypeptide thymosin α-1 (Tα-1) rescues CFTR maturation, stability, and activity, thereby correcting the pathological abnormalities of F508del-CFTR *in vitro* (in CFBE41o- cells and in primary human bronchial epithelial cells), and *in vivo* in a CF murine model^38^. Tα-1 was also reported to increase expression of the calcium-activated chloride channel (CaCC) regulator CLCA1 in three of five patients examined, and proposed to promote compensatory chloride secretion^38^. These multiple activities of Tα-1 were attributed to the same mechanism of action as the previously reported anti-inflammatory and immunomodulatory effects of Tα-1, by elevating indoleamine 2,3-dioxygenase 1 (IDO1) expression in the bronchial epithelium^38^. Indeed, by increasing IDO1 expression, Tα-1 reduces proinflammatory signaling and initiates immunotolerance in the lung^38^. Considering that IDO1 is a potent driver of autophagy, Tα-1 was proposed to restore autophagy in CF cells and rescue CFTR^38^, similarly to cysteamine^37^. Tα-1 could therefore be considered a prototypic single-molecule-based therapy for CF.

The enthusiasm for these findings however waned when three independent laboratories were unable to reproduce the results^39,40^. Romani and colleagues replied to these studies by calling into question the acetylation status and handling of the Tα-1 peptide, in particular the vehicle used^41^. Considering that a single-molecule-based therapy would be of great importance for CF patients, here we directly addressed these potential issues and to clarify whether indeed Tα-1 has any impact on CFTR trafficking or function.

## Results and Discussion

Our previous studies conducted in three independent laboratories failed to detect Tα-1 activity as modulator of mutant CFTR maturation and function on primary bronchial epithelia from F508del homozygous patients by using biochemical and electrophysiological techniques^39,40^. These results were obtained by testing Tα-1 from two different commercial sources (CRIBI and Abcam) and using different protocols for peptide solubilization (Tα-1 powders were dissolved in either distilled water, DMSO or 0.1% acetic acid). An independent laboratory confirmed the sequence of the synthetic Tα-1 peptides using LC-MS/MS sequence analysis^39^. Given the unexpected negative results, the biological activity of Tα-1 was confirmed by assessing its ability to induce apoptosis in MCF-7 breast cancer cells^39^. Romani and colleagues replied to these studies by calling into question the acetylation status of the Tα-1 peptides and their mode of solubilization^41^. Indeed, they reported that Tα-1 undergoes remarkable and permanent structural changes when dissolved initially in DMSO leading to peptide aggregation that cannot be reversed by subsequent dilution in aqueous medium^41^. However, data supporting these claims regarding the vehicle dependence of Tα-1 were not presented. In addition, they suggested that the use of Tα-1 peptide purchased from Abcam, being non acetylated at the N-terminus, was perhaps the main reason for the lack of F508del-CFTR correction by Tα-1, since acetylation of Tα-1 is important for its biological activity^41^. In agreement, they found that only the CRIBI peptide, being acetylated, was active on CFTR^41^.

### Evaluation of Tα-1 sequence, N-terminus acetylation and native conformation

**Figure 1:**
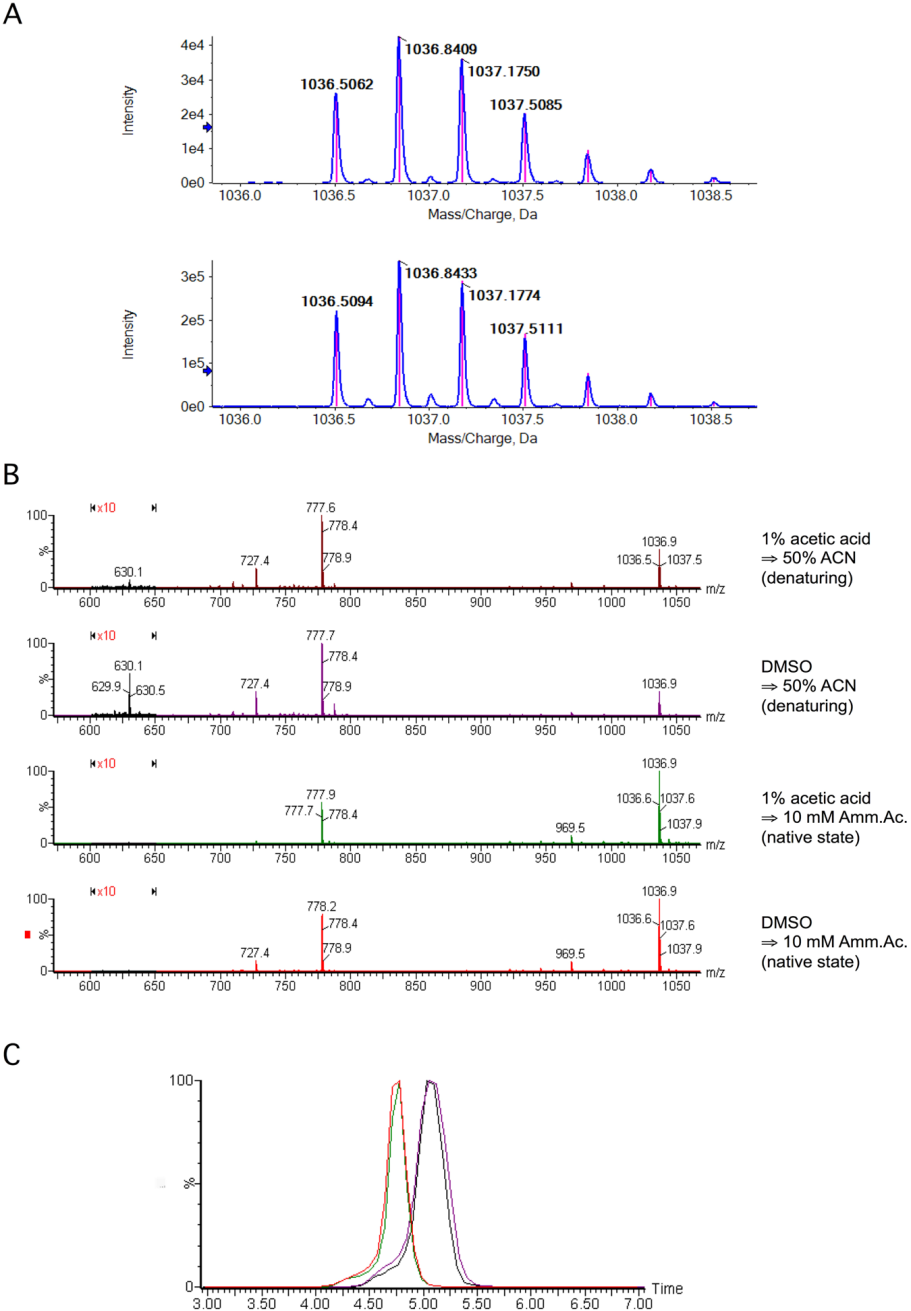
MS, MS/MS and Ion Mobility analysis of thymosin alpha-1 peptide. (**A**) High-resolution MS spectra of both Abcam (top) and CRIBI (bottom) peptides, measured for charge state +3. The plot shows the measured mass spectrum (blue line) and the overlapped theoretical isotopic profile for the calculated brute formula C_129_H_215_N_33_O_55_ with charge state 3. This brute formula differs from the one calculated for native Tα-1 (C_127_H_213_N_33_O_54_) for one acetyl group (CH3-CO-). This confirms that both peptides are acetylated. (**B**) MS spectra for 4.18 μM Tα-1. In panels A and B, peptide was initially dissolved in 1% acetic acid (Panel A) or DMSO (Panel B) and then diluted 1000X in 50% ACN, pH 2 (denaturing conditions). In panels C and D, the peptide was again dissolved in 1% acetic acid (Panel C) or DMSO (Panel D) but then diluted 1000X in 10 mM ammonium acetate, pH 7.4 (native state conditions). Charge states 3+ (1037 m/z) and 4+ (778 m/z) are visible in both native and denaturing conditions, although with different relative intensities. By contrast, charge state 5+ (630.1 m/z) is only visible in denaturing conditions (Panels A and B), thus indicating the loss of native 3D conformation of Tα-1. (**C**) IMS analysis of Tα-1. Mobilograms (drift time Vs ion intensity plots) for charge state 3+ of Tα-1 a brought to native conditions from 1% acetic acid (red) or DMSO stocks (green) and brought to denaturing conditions from 1% acetic acid (blue) or DMSO stocks (purple).

Our previous analyses however demonstrated that *both* Tα-1 peptides (Abcam and CRIBI) *were acetylated* at the N-terminus^39^. Although not mentioned on the Abcam website, the Certificate of Compliance for their Tα-1 peptide clearly indicates that the peptide is indeed acetylated (available upon request). We further investigated the acetylation status of the Tα-1 peptides by performing our own high-resolution mass-spectrometry analysis of both peptides. Figure 1A shows the mass-to-charge ratio observed for charge state +3 for both samples.

The observed mass for both peptides (Abcam and CRIBI) perfectly matches that predicted for Tα-1 bearing one acetyl group. Unlike Romani *et al*. we also confirmed in-house, by using MS/MS analysis, that (1) the observed sequence matches the native one and that (2) the acetyl group is at the N-terminus, as reported in Supplementary Figs 1 and 2. Motivated by the suggestion that the initial dissolution conditions used to prepare Tα-1 stock solutions could have a dramatic and non-reversible effect on its biological activity after aqueous dilution^41^, we carried out experiments to test this possibility. Indeed, it was difficult that a relatively short, predominantly alpha helical peptide of 28 amino acids could be irreversibly altered by the initial dissolution conditions (Supplementary Fig. 3).

We then explored Tα-1 3D structure by native state MS^42,43^ coupled to ion mobility MS (IMS). This well established technique accurately monitors changes in the 3D structures of peptides and proteins^44^. Biomolecules are introduced into the spectrometer dissolved in a MS-compatible buffer (e.g. 10 mM ammonium acetate, pH 7.4) that does not alter their native state conformation. The electrospray process (ESI+) transfers positively charged molecules from the liquid to the gas-phase. During the IMS analysis, these ions move through a drift cell filled with an inert gas (N_2_) before they reach the time of flight (ToF) mass analyzer. Ions having the same mass-to-charge ratio (m/z) but different 3D conformations are transmitted through the drift cell at slightly different velocities, thus generating an effective gas-phase separation measured in milliseconds (Drift Time). From the drift time values, a collisional cross section (CCS, in Å^2^) can be calculated for each ion after calibration with reference compounds of known CCS values.

We analyzed by IMS Tα-1 samples prepared from both a DMSO stock solution and from a 1% acetic acid stock solution, looking for differences in their DT values that might indicate changes in their 3D conformations. Subsequent dilutions by 1000-fold under native state (10 mM ammonium acetate pH 7.4) and denaturing conditions (H_2_O/CH_3_CN 50/50 + 0.1% HCOOH, pH = 2.5) yielded four samples: Tα-1_DMSO,native_, Tα-1_DMSO,denat_, Tα-1_Acetic,native_ and Tα-1_Acetic,denat_. The final peptide concentration of each sample was 4.18 μM. As shown in Fig. 1B, the mass spectra of Tα-1 in denatured or native conditions differed significantly.

More ionizable residues were exposed to solvent and thus to protonation in denaturing conditions (50% organic solvent, pH 2), resulting in higher intensities of the higher charge states (4+, 778 m/z) compared to lower ones (3+, 1037 m/z). The predominantly α-helical structure of the peptide (see Supplementary Fig. 3) was altered in denaturing conditions, so that even charge state 5+ (630 m/z) became visible (denatured state). When the 3D structure was maintained (native state), the greatest intensity was for lower charge states (less polar residues were exposed to the solvent). This indicates that these denaturing conditions abolish the original native state 3D structure. We then activated the ion mobility device of the MS and measured the DT values for each of the four samples. Figure 1C reports the mobilograms for each Tα-1 sample, i.e. plots of the measured intensity of the triply charged ion (1036 m/z) vs the observed drift time value.

Tα-1 unfolded in denaturing conditions, as demonstrated by the increased DT value observed for Tα-1_DMSO,denat_ and Tα-1_Acetic,denat_. Although this difference (~0.4 msec) was clearly detected by our setup, it was not a large change, indicating that the original 3D native structure of Tα-1 may be energetically similar to the disordered one in denaturing conditions. Indeed, Tα-1 structure has been previously investigated by NMR spectroscopy under non-standard (60% trifluoroethanol in water) conditions^45^. It is important to point out here that no traces of aggregated states of Tα-1, visible as highly charged polymers, were observed by MS, in any of the four tested conditions (DMSO/acetic acid – native state/denaturing conditions). NMR and molecular dynamics indicate Tα-1 in aqueous solutions forms two short β turns at the N-terminus, followed by a short α-helix (residues 14–26)^45^. More importantly, when Tα-1 was returned to native conditions for MS, no differences in DT were observed between samples derived from the two different stock solutions. This demonstrates that Tα-1 has no “memory” of its original dissolution conditions (as expected after 1000X dilution). Any change in Tα-1 induced by the solvent used for the stock solution was fully reversible and the peptide effectively reverted to its native state when diluted for biological tests. To further characterize Tα-1 conformational states, we also converted the observed DT values into CCS values by using myoglobin as a calibration^44^. Table 1 reports the measured CCS values for the four Tα-1 conditions and for charge states 2+, 3+ and 4+.

**Table 1:** Calculated collisional cross section values (CCS) in Å^2^ for each of the four Tα-1 samples at charge states 2+, 3+ and 4+. CCS calibration was done using myoglobin as reference compound.

m/z	1554.36	1036.57	778.18
Charge State	2+	3+	4+
Tα-1_DMSO,native_	454.5	491.7	529.8
Tα-1_DMSO,denat._	472.9	509.2	551.1
Tα-1_Acetic,native_	454.4	491.1	530.0
Tα-1_Acetic,denat_.	477.7	512.6	555.4

The CCS values we calculated for Tα-1 are comparable to those calculated previously by other groups for similar peptides, such as Neuropeptide Y (2658 Da, CCS 417 Å^2^) and Melittin (2848 Da, CCS 469 Å^2^) ^46^.

### Evaluation of pro-apoptotic activity of Tα-1 peptides following different solubilization protocols

**Figure 2:**
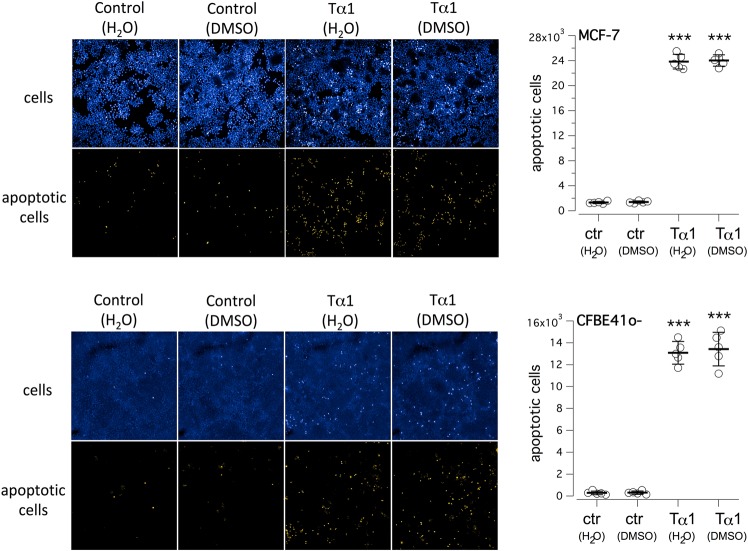
Evaluation of thymosin α-1 effect on induction of cell apoptosis. (**A**) Analysis by means of high-content confocal imaging of MCF-7 breast cancer cells following 72-hour treatment with Tα-1 (100 µM) pre-diluted as 200X stock in DMSO or ddH_2_O. (**B**) Dot-plot reporting quantification of apoptotic MCF-7 breast cancer cells treated as in A. (**C**) High-content confocal image analysis of CFBE41o- bronchial epithelial cells after 72-hour treatment with Tα-1 (100 µM) as in A. (**D**) Dot-plot showing quantification of the number of apoptotic CFBE41o-cells treated as in A. Mean values ± SD are shown (*n* = 5). ***P < 0.001 versus respective negative control by ANOVA.

To assess the biological activity of Tα-1 prepared using different solubilization protocols, we evaluated its ability to induce apoptosis in MCF-7, a breast cancer cell line, after long-term treatment at high-micromolar concentration, as already reported by several groups^47,48^. MCF-7 cells were plated at low density on high-quality 96-well plates appropriate for confocal imaging and, after 6 hr, they were treated with Tα-1 (100 µM) from 200X stocks prepared in either DMSO or ddH_2_O. Then, cell nuclei were counterstained with Hoechst 33342 and propidium iodide to discriminate between living and apoptotic cells (Fig. 2A). We observed a more than10-fold increase in the number of apoptotic cells upon treatment with Tα-1, with no differences between cells treated with peptide diluted from DMSO or ddH_2_O stock solutions (Fig. 2B). We then evaluated whether a similar pro-apoptotic activity was also observed in bronchial epithelial cells, the target tissue for the corrector activity of Tα-1. The effect of Tα-1 (100 µM) on immortalized bronchial epithelial CFBE41o- cells was similar to that on MCF-7 cells. Tα-1 peptides from either DMSO or ddH_2_O stocks had marked pro-apoptotic activity, causing a >50-fold increase in the number of apoptotic nuclei (Fig. 2C,D). These results demonstrate that Tα-1 peptides prepared from the two different stock solutions had equivalent biological activity when monitored as apoptosis inducer.

### Tα-1 does not rescue F508del-CFTR activity or expression in well-differentiated human bronchial epithelia derived from CF patients as evidenced by electrophysiological and biochemical studies

The ability of Tα-1 to correct defective processing of F508del-CFTR was evaluated by multiple independent, established laboratories working in the CF field using a variety of standard protocols. Tα-1 was tested, using electrophysiological techniques, on well-differentiated human bronchial epithelia, derived from F508del homozygous subjects.

Bronchial cells were seeded on permeable supports and cultured under air-liquid interface (ALI) conditions until they were well differentiated. The protocols followed for cell culture media and other methods were those routinely utilized in each laboratory. As positive control, all the laboratories benchmarked responses to those produced by VX-809^14^. Since cysteamine is thought to share the same mechanism of action as Tα-1^38^, some laboratories also included cysteamine in their tests alone or in combination with epigallocatechin gallate (EGCG), which has been reported to prolong cysteamine effects on mutant CFTR rescue^37^.

**Figure 3:**
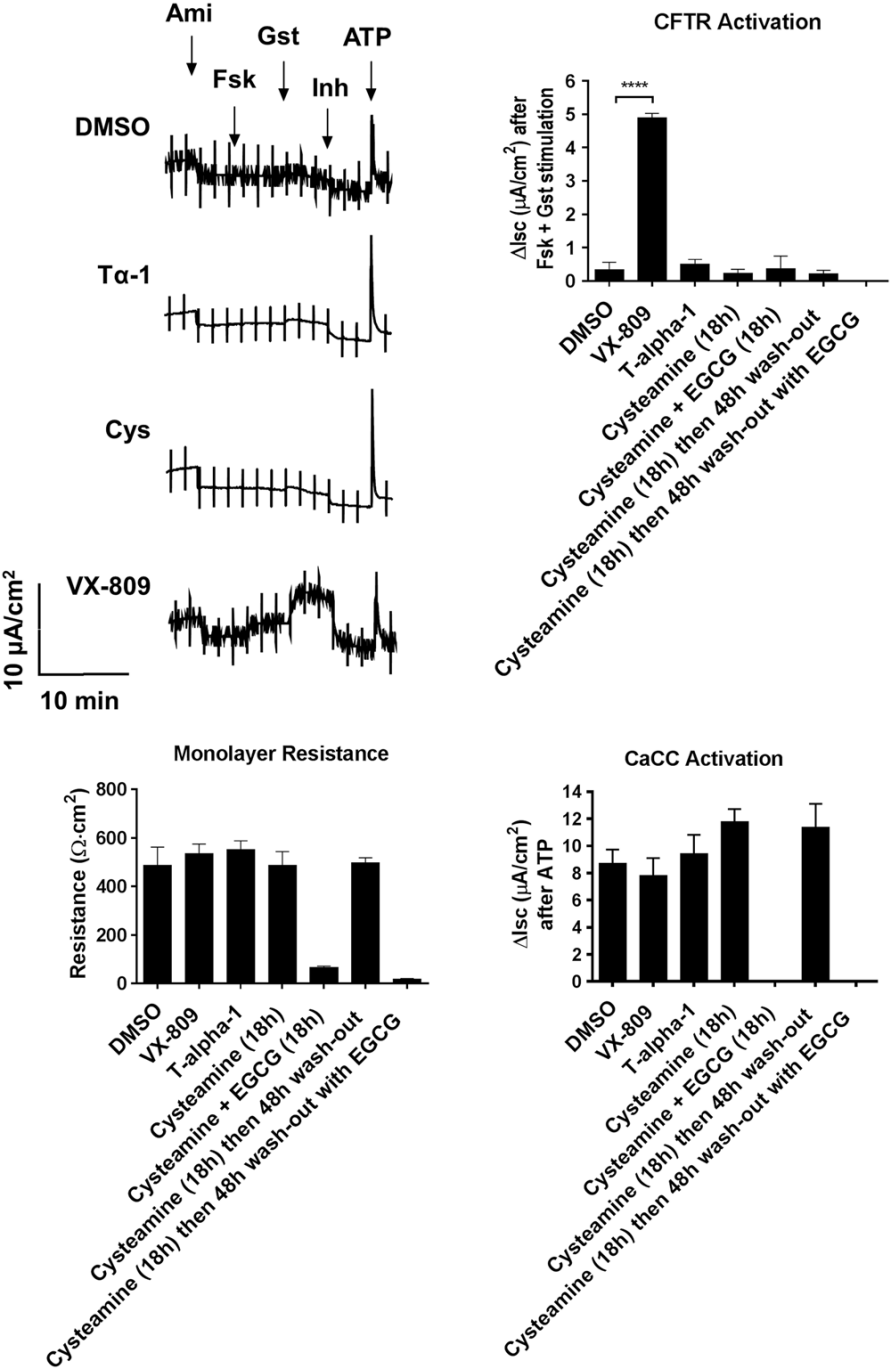
Thymosin α-1 (Tα-1) effect on human bronchial epithelia (HBE) derived from F508del-CFTR homozygous patients. Representative traces of short-circuit current measurements performed on HBE epithelia derived from a homozygous F508del patient were assayed upon a 24-hour treatment with DMSO alone (0.1%), Tα-1 (100 ng/ml + 0.1% DMSO), VX-809 (3 μM), cysteamine (250 μM) or cysteamine plus EGCG (80 μM). The bar graphs show the corresponding monolayer resistance, CFTR-mediated currents and CaCC-mediated currents Mean values ± SD are shown (*n* = 3–6). ****P < 0.0001 versus negative control by ANOVA.

In the first laboratory (John Hanrahan and colleagues, McGill University, Montreal, Canada), well differentiated human bronchial epithelial (HBE) cells from a F508del-CFTR homozygous patient were cultured in complete ALI medium for 4 weeks and switched to bovine pituitary extract-free ALI medium 48 h before measurements. Cells were treated in this medium by adding DMSO (0.1% as negative control), VX-809 (1 µM), Tα-1 (100 ng/mL, previously dissolved in H_2_O), cysteamine (250 µM, previously dissolved in H_2_O), EGCG (80 µM, dissolved in DMSO) and combinations of these compounds as indicated in Fig. 3. All treatments were begun 24 h or 18 h prior to Ussing Chamber measurements except for cysteamine wash-out, in which cells were treated for 18 h followed by a 48 h wash-out in the presence or absence of EGCG. After treatments, cells were mounted in Ussing Chambers with a basolateral-to-apical Cl^−^ gradient. Amiloride (10 µM apical) was added on the apical side to inhibit Na^+^ currents carried by ENaC, and CFTR currents were measured after adding forskolin (10 µM) and genistein (50 µM) on the apical side. CFTR_inh_-172 (10 µM) was then added on the apical side to inhibit CFTR-dependent current followed by apical ATP (100 µM) addition to activate transient Ca^2+^-activated Cl^−^ current as a positive control and to confirm cell viability. As expected, incubation with VX-809 resulted in significant F508del-CFTR rescue, as evidenced by the relatively large current increase elicited by the addition of forskolin and genistein and the amplitude of the block caused by CFTR_inh_-172 (Fig. 3A). No differences in CFTR-mediated currents were observed in epithelia treated with Tα-1, cysteamine or cysteamine + EGCG (Fig. 3A). None of the treatments influenced the amplitude of the ATP-stimulated current, which monitors activation of CaCCs (Fig. 3A). Incubation with cysteamine + EGCG caused a marked decrease in transepithelial resistance, suggesting that this treatment compromises the integrity of the epithelial culture (Fig. 3B).

**Figure 4:**
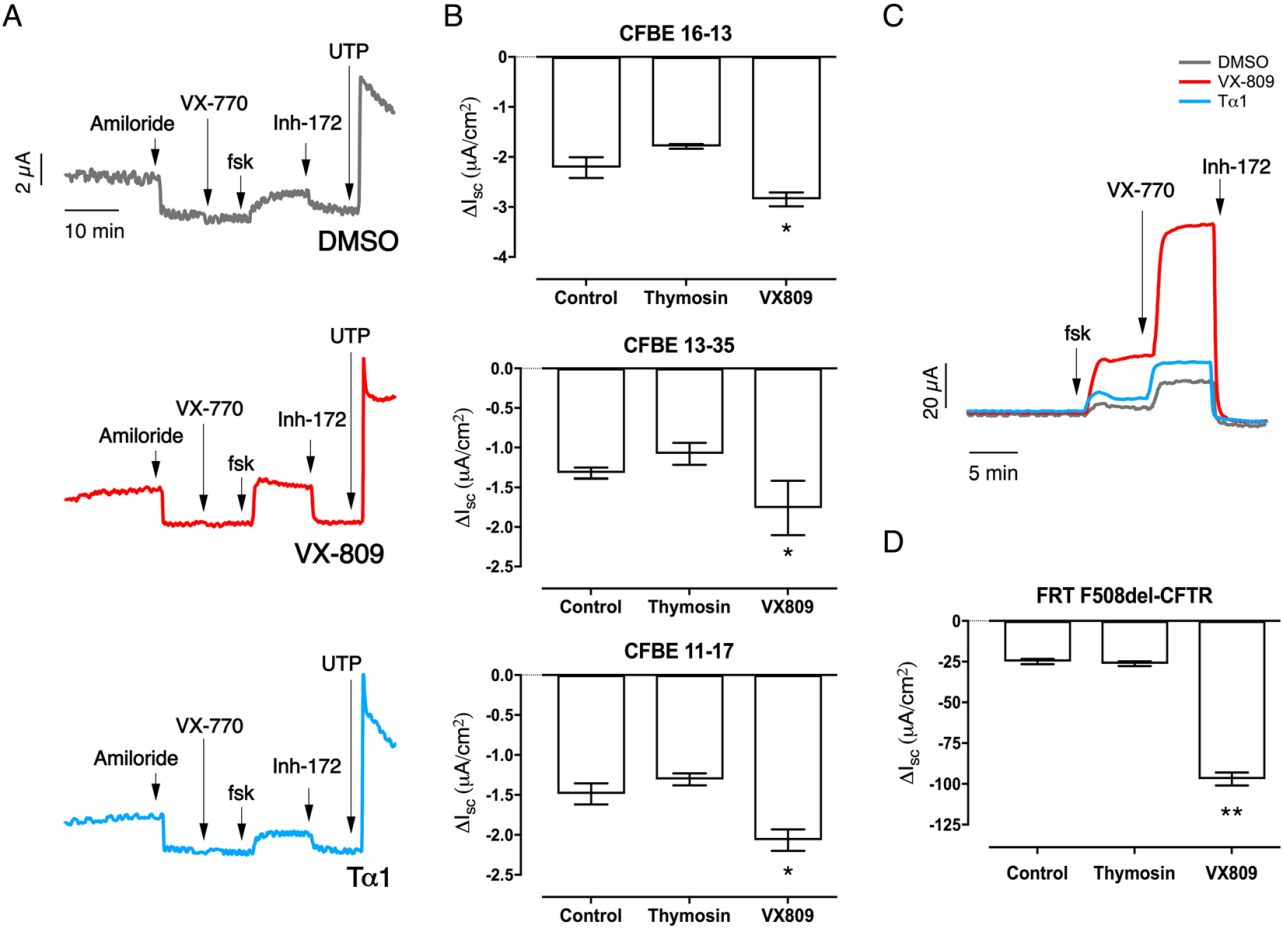
Evaluation of Tα-1 effect as CFTR modulator on human primary bronchial epithelia (HBE) derived from CF patients and on F508del-CFTR-expressing FRT cells. (**A**) Representative traces of short-circuit current measurements performed on HBE derived from a homozygous F508del patient (donor code CFBE 13–35) after 24-hour treatment with vehicle alone (0.1% DMSO), Tα-1 (100 ng/ml), or VX-809 (3 μM). (**B**) Bar graphs reporting CFTR-mediated currents as measured during Ussing chamber recordings of HBE (generated from cultures derived from 3 homozygous F508del patients) treated as described in A. (**C**) Representative traces obtained during Ussing chamber recordings of FRT epithelia treated as in A. (**D**) Bar graphs summarizing CFTR-mediated currents derived from experiments as those described in A. Data are means ± SD (*n* = 3–6). **P < 0.01, *P < 0.05 versus negative control by ANOVA.

In the second laboratory (Alan S. Verkman and collaborators, University of California San Francisco), well differentiated bronchial epithelia from three different F508del/F508del CF patients were incubated with 3 μM VX-809, or 100 ng/mL human Tα-1 peptide, or control, at the basolateral side for 18–24 hours at 37 °C prior to measurements. Changes in short-circuit current (I_sc_) were then recorded in Ussing chambers after ENaC block by amiloride, CFTR activation by forskolin and the potentiator VX-770 (1 µM) and finally CFTR inhibition by CFTR_inh_-172. Currents mediated by F508del-CFTR were not affected significantly by Tα-1 pre-treatment whereas those exposed to VX-809 displayed a significant increase in CFTR-dependent function (Fig. 4A,B). The ability of Tα-1 to rescue mutant CFTR function was also tested using Fischer Rat Thyroid (FRT) cells stably expressing F508del-CFTR (Fig. 4C). This cell line has been extensively used for correctors studies^49,50^. Similarly to what was observed with primary bronchial epithelia, VX-809, but not Tα-1, produced a significant increase in CFTR function (Fig. 4C,D).

**Figure 5:**
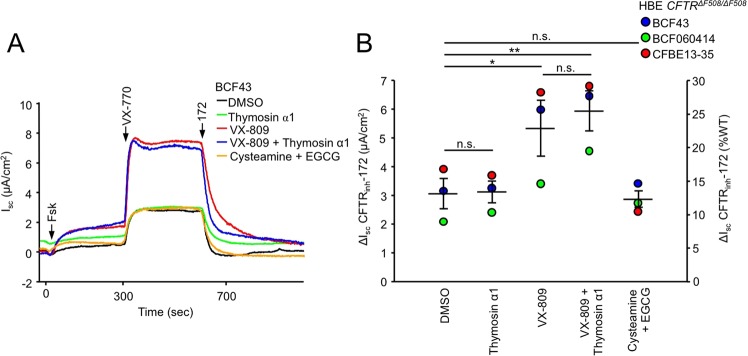
Thymosin α1 or cysteamine + EGCG do not improve F508del-CFTR function in primary human bronchial epithelia. (a) Effect of 24 hours treatment with thymosin α1 (100 ng/ml), VX-809 (3 μM), cysteamine (250 μM) + EGCG (80 μM) or combinations on the I_sc_ of human bronchial epithelia with *CFTR*^*F508del/F508del*^ genotype (CF-HBE). Short-circuit currents were recorded in an intact monolayer with equimolar chloride concentrations in both chambers. CFTR-mediated currents were induced by sequential stimulation with forskolin (Fsk, 20 μM) and VX-770 (3 µM) followed by CFTRinh-172 (172, 20 µM) to completely inhibit CFTR (b) Quantification of the CFTR_inh_-172 sensitive current in CF-HBE isolated from three homozygous F508del CF patients after single or combination treatment shown as μA/cm^2^ (left axis) or expressed as percentage of the mean WT-CFTR currents measured in WT-HBE, isolated from five donors (right axis)^20^. N.s. - not significant, *P < 0.05, **P < 0.01 by paired two-tailed Student’s t-test. Data are means ± SEM.

In the laboratory headed by Gergely Lukacs (McGill University, Montreal, Canada), the efficacy of Tα-1 (stock reconstituted in 0.1% acetic acid) and cysteamine (stock freshly solubilised in aqueous solution) plus EGCG (stock solubilised in ethanol) was evaluated on *CFTR*^*F508del/F508del*^ human bronchial epithelia derived from three subjects. Quantification of the CFTR-mediated currents, elicited by sequential addition of forskolin and VX-770 followed by CFTR inhibition with CFTR_inh_-172, showed that only treatment with VX-809 resulted in significant F508del-CFTR rescue (Fig. 5).

**Figure 6:**
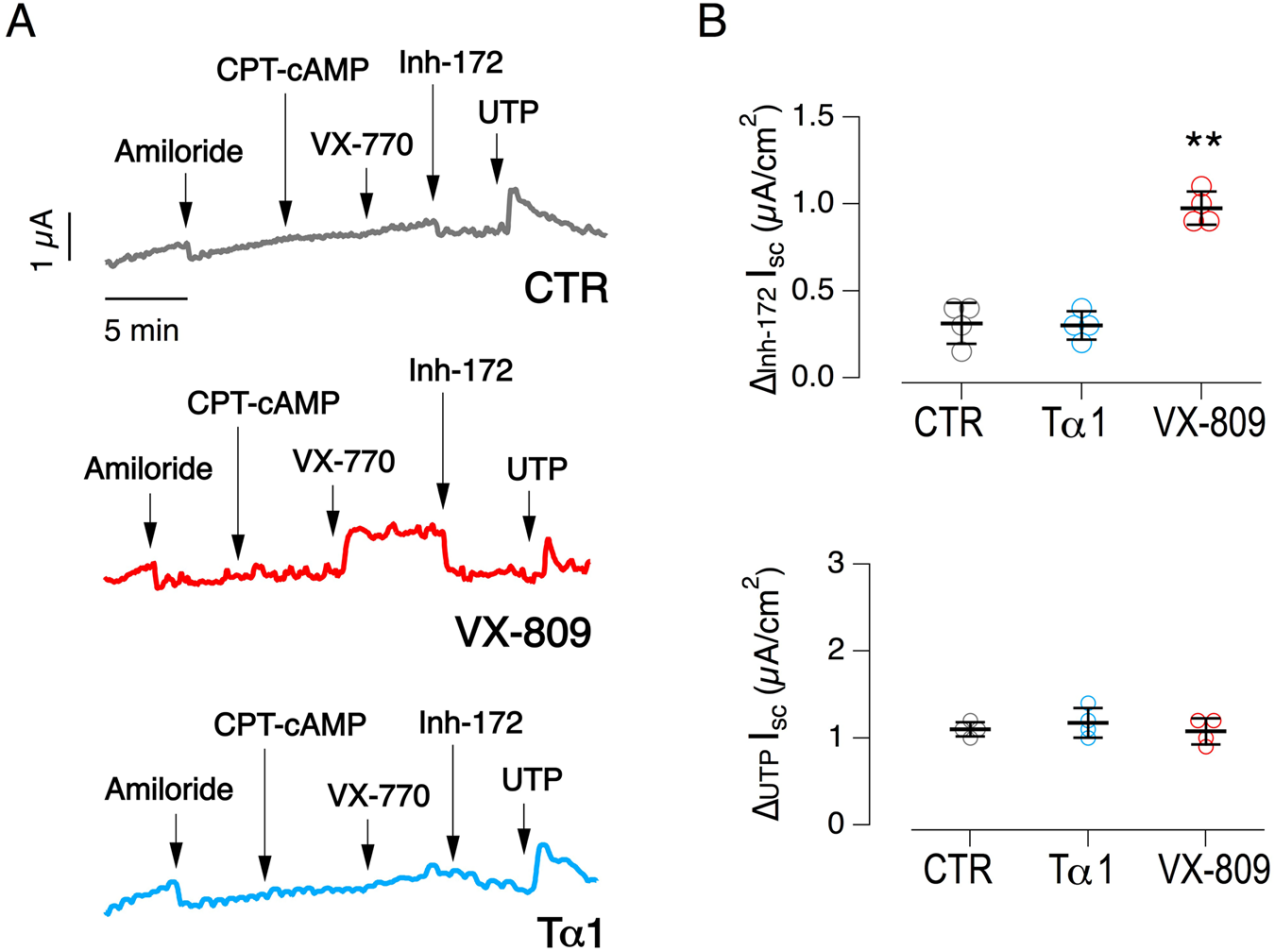
Evaluation of thymosin α-1 as F508del-CFTR rescue maneuver on human CF primary bronchial epithelia (HBE). (**A**) Representative traces obtained from Ussing chamber experiments on HBE generated from a homozygous F508del patient (donor code CF-BEX05), after incubation for 24-hour with vehicle, Tα-1 (100 ng/ml, pre-diluted in acqueous buffer), VX-809 (3 μM). (**B**) Dot plot reporting CFTR-mediated (top) and CaCC-mediated (bottom) currents measured during the experiments depicted in (**A)**. Mean values ± SD are shown (*n* = 4). **P < 0.01 versus negative control by ANOVA.

Similar experiments were performed also by Luis J.V. Galietta and collaborators (Tigem, Pozzuoli, Italy). Primary bronchial epithelia from one F508del homozygous patient were treated with Tα-1, vehicle (DMSO) and VX-809. Also in this laboratory, no increased CFTR-activity was observed in Tα-1-treated epithelia, while VX-809 elicited a nearly 3-fold increase in the amplitude of CFTR-mediated current (Fig. 6). Similarly, Tα-1 treatment did not alter CaCC-mediated current elicited by UTP addition (Fig. 6).

**Figure 7:**
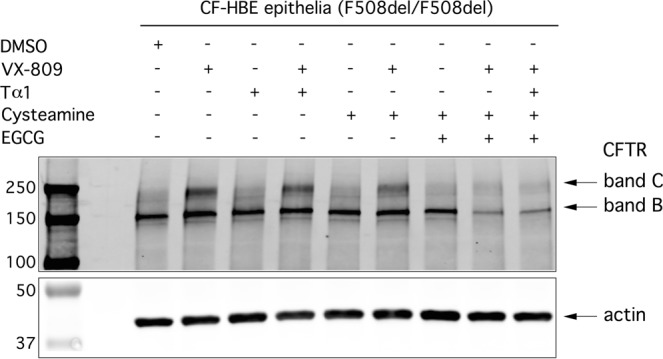
Biochemical evaluation of the effect of Tα-1 on mutant CFTR expression pattern in HBE from a F508del homozygous patient. Epithelia were treated with the indicated compounds for 48 hours. CFTR was immunoprecipitated from lysates using rabbit anti-CFTR antibody 155 and then detected by Western blot analysis with antibody CFFT-596. The mature, complex-glycosylated (band C) and the immature, core-glycosylated (band B) forms of CFTR protein are indicated by arrows. Full-length (uncropped) blots/gels are presented in Supplementary File 1.

Finally, by using biochemical techniques, Martina Gentzsch and colleagues, from the University of North Carolina at Chapel Hill, investigated mutant CFTR maturation in primary F508del/F508del bronchial epithelia, following treatment for 48 h with vehicle, VX-809 (5 µM), Tα-1, cysteamine, and EGCG, alone or in combination. After treatment, cells were lysed, separated by SDS-PAGE, and analyzed by immunoblotting (Fig. 7). CFTR protein bands of ~150 and 170 kDa were detected, corresponding to the immature core-glycosylated band B form and mature, complex-glycosylated band C, respectively. F508del-CFTR lysates contained primarily band B, as consistent with the severe trafficking defect caused by this mutation. In agreement with functional data, treatment with VX-809 increased expression of the mature band C form when compared with vehicle control conditions (DMSO; see Fig. 7). However, no changes in the appearance of band C were detected following treatment of cells with Tα-1, cysteamine or EGCG, either individually or in combination (Fig. 7). Of note, when epithelia were pretreated with the triple combination of VX-809 + cysteamine + EGCG, CFTR expression was greatly reduced and there was no increase with VX-809 (Fig. 7). Similar results were recently reported by the group of Margarida Amaral, in a study focused on the R560S-CFTR mutant^51^.

## Conclusions

The results reported in this study from six independent CF research laboratories, together with the negative data published earlier^39,40^, fail to demonstrate any correction of the F508del-CFTR by Tα-1, regardless of the solvent used to dissolve the peptide. Despite using different bronchial cell culture and measurement protocols, all laboratories found that the corrector VX-809 consistently produced a significant increase in CFTR-mediated chloride secretion, which was paralleled by the appearance of the mature form of the CFTR protein as visualized by immunoblot analysis. Although not an exhaustive assessment, this study also calls into question the activity of the autophagy modulator cysteamine, and its combination with the antioxidant food supplement EGCG. Indeed, not only was rescue of mutant CFTR function not detected following 24–48 h treatment with cysteamine, but there appeared to be deleterious effects on CFTR expression and activity when bronchial epithelia were exposed to cysteamine + EGCG. Although these studies do not exclude the possibility of beneficial immunomodulatory effects of thymosin α-1, they do not support its utility as a corrector of F508del-CFTR.

The pharmacotherapy of the basic defect in CF, i.e. the rescue of the mutant CFTR protein with small molecules, has become a reality for patients, particularly for those carrying the severe and frequent F508del mutation. Combinations of a corrector and a potentiator, e.g. VX-809 and VX-770^15^, to address F508del-CFTR trafficking and gating defects, have been already approved. These combinations have a relatively modest clinical effect, probably because the mutation causes multiple defects in CFTR protein folding and stability^18^. It is established that better results can be achieved by combining correctors having complementary mechanisms of action^18,20^. In this respect, many clinical trials including combinations of correctors are under way. Recently, two studies have demonstrated a remarkable improvement of clinical condition in patients with one or two copies of F508del by using two correctors together^52,53^. Importantly, the same studies reported strong synergistic effects of these corrector combinations on F508del-CFTR function and trafficking *in vitro*, thus providing scientific evidence for the effects observed *in vivo*. The present findings highlight the importance of advancing to clinical trials only those drugs for which there is solid scientific evidence of efficacy. This will become increasingly important if the availability of patients willing to participate in future clinical trials declines as they progress to stable use of approved CFTR modulators. Only drugs with strong evidence of improved efficacy compared to available treatments should be considered. The results obtained so far by multiple laboratories do not support evaluation of the autophagy modulators Tα-1 and cysteamine in clinical trials.

## Methods

### Chemicals, reagents and analytical standards for LC-MS/MS analysis

Chemicals and reagents used for sample preparation and LC-MS/MS analysis were purchased from Aldrich (Milano, Italy). Thymosin α-1 was purchased from CRIBI (CRIBI Biotechnology Center, Peptide Facility, Università di Padova, Italy) and from Abcam (cat. #ab42247). The peptides were acetylated at the N-terminus, as stated in the Vendors’ specifications and as confirmed by in-house MS analysis. The exact sequence, including the acetylation, was checked by tandem mass spectrometry and confirmed.

### High-resolution intact mass and MS/MS analysis

The analysis was performed as previously detailed^39^. In brief, synthetic Tα-1 powders were dissolved at 2 mg/ml in either DMSO or 0.1% acetic acid. Samples were then diluted to 10 microgram/ml in 3% acetonitrile in water. Two microliters of each solution were then loaded on a nanoAcquity UPLC system (Waters Inc) coupled to a 5600+ TripleToF mass spectrometer (SCIEX). The samples were desalted on a 180 μm × 20 mm nanoAcquity trapping column (Waters Inc) and then moved to a 75 μm × 250 mm Picofrit column (Scientific Instruments Service). Eluents were A (water +0.1% formic acid) and B (acetronitrile +0.1% formic acid). Flow rate was set to 300 nl/min. The samples were eluted from the column with a linear gradient of B from 3% to 30% in 15 minutes. Peptides were analyzed in positive nanospray ion mode. Scan range was set from 400 to 1,500 m/z for MS and from 100 to 1,800 m/z for MS/MS. Charge states 3 and 4 precursors were selected for MS/MS. Collision energy profiles were set according to SCIEX settings.

### Ion mobility mass spectrometry analysis

Two reference standards of 4.18 mM were prepared by dissolving Tα-1 in pure DMSO or 1% acetic acid. Tα-1 was perfectly soluble in both solutions. The two stock solutions were then diluted to 4.18 μM (1000X) in either 10 mM CH_3_COONH_4_, with pH adjusted to pH = 7.4 (for native state MS) or H_2_O/CH_3_CN 50/50 + 0.1% HCOOH, pH = 2.5 (for denatured state MS). This generated four final solutions; i.e. the DMSO and aqueous stock solutions and the final dilutions corresponding to native and denaturing conditions (Tα-1_DMSO,native_, Tα-1_DMSO,denat_, Tα-1_Acetic,native_ and Tα-1_Acetic,denat_, respectively). These solutions were infused at 5 μl/min. into a Synapt G2 mass spectrometer (Waters, Milford, MA, USA) operating in positive electrospray mode and with the ion mobility feature enabled. Capillary was set to 3 kV, sampling cone at 30 V, cone gas flow was set to 50 l/hour, gas and source temperatures were set to 450 °C and 90 °C respectively. IMS gas flow was set to 90 ml/min. IMS wave height was set to 40 V and wave velocity was set to 600 m/s. A 0.1 mg/ml solution of myoglobin in water was used as calibrator to convert the observed delay time (DT) into collisional cross section (CCS) values, The reference values calculated by Ruotolo *et al*.^44^ for myoglobin were used. Masslynx and DriftScope software (from Waters) were used for data analysis.

### Reagents for biological studies

Gaslini Laboratory: Tα-1 peptides were purchased from CRIBI (CRIBI Biotechnology Center, Peptide Facility, Università di Padova, Italy) and from Abcam (cat. #ab42247). Thymosin α1 peptides were dissolved at 2 mg/ml in either DMSO or ddH_2_O, VX-809 (SelleckChem, S1565) was solubilised in DMSO (10 mM), cysteamine (Sigma, M9768) was freshly solubilised in aqueous solution at the desired final concentration.

McGill Laboratory: Thymosin α1 (abcam, ab42247) was reconstituted in 0.1% acetic acid, VX-809 (Selleck, S1565) was solubilised in DMSO, cysteamine (Sigma, M9768) was freshly solubilised in aqueous solution, and epigallocatechin gallate (EGCG, Sigma, E4143) was solubilised in ethanol.

UCSF Laboratory: VX-809, VX-770 and CFTRinh-172 were purchased from Selleck Chemicals (Boston, MA). Human Thymosin alpha-1 peptide (ab42247) were purchased from Abcam (Cambridge, MA).

### Culture of cell lines

CFBE41o- cells were cultured in MEM medium supplemented with 10% FCS, 2 mM L-glutamine, 100 U/ml penicillin, and 100 μg/ml streptomycin.

MCF-7 cells (human breast adenocarcinoma cell line; ATCC) were cultured in DMEM medium supplemented with 10% FCS, 2 mM L-glutamine, 100 μg/ml streptomycin and 100 U/ml penicillin.

Fischer rat thyroid (FRT) cells expressing F508del-CFTR were described previously^50^ and cultured in Kaighn’s modified Ham’s F-12 medium supplemented with 10% FBS, 2 mM l-glutamine, 100 μg/mL streptomycin, 100 units/mL penicillin, 18 μg/mL myoinositol, and 45 μg/mL ascorbic acid.

### Evaluation of apoptosis in cell lines

The analysis was performed as previously detailed^39^. In brief, MCF-7 or CFBE41o- cells were plated at low density (10,000 cells/well) on high-quality 96-well plates suitable for high-content imaging. Six hours after plating, cells were treated with Tα-1 (100 µM) prepared from 200X DMSO or aqueous stocks. After 72 hours, Hoechst 33342 and propidium iodide were used to counterstain cells, in order to visualize total cells and apoptotic cells, respectively. Plates were imaged using an Opera Phenix (PerkinElmer) high-content imaging system, using a ×20 air objective. The excitation of Hoechst 33342 signal was at 405 nm and the emission at 435–480 nm. The propidium iodide signal was excited at 560 nm and the emission was measured at 570–630 nm.

### Primary bronchial epithelial cell culture

#### McGill Laboratory – Hanrahan

Well differentiated HBE cells from a F508del-CFTR homozygous patient were cultured in complete ALI medium for 4 weeks and switched to bovine pituitary extract-free ALI medium 48 h before measurements. Cells were treated in this medium by adding DMSO (0.1% as negative control), VX-809 (1 μM), Tα-1 (100 ng/mL, previously dissolved in H_2_O), cysteamine (250 μM, previously dissolved in H_2_O), EGCG (80 μM, dissolved in DMSO) and combinations of these compounds as indicated in the figure. All treatments began 24 h or 18 h before Ussing chamber measurements except for those involving cysteamine wash-out, in which cells were treated for 18 h followed by a 48 h wash-out period in the absence or presence of EGCG.

#### UCSF Laboratory

Primary human F508del/F508del CF bronchial epithelial cells were isolated from lung explants and cultured on semipermeable inserts (Snapwell, Corning Inc.) in ALI media at an air-liquid interface, as described^54^. The medium was changed every 2–3 days. Conditional reprogramming on two F508del/F508del CF subjects primary bronchial epithelial cells was performed as described^55^. All protocols involving the collection and use of human tissues and cells were reviewed and approved by the University of California, San Francisco Institutional Review Board.

#### UNC Laboratory

Bronchial epithelia cells were cultured as previously described^56^ and seeded on Millicell inserts (12-mm Millipore inserts PICM01250) and maintained at air-liquid interface for 18 days in serum-free BEBM/DMEM (1:1)–based medium^57^ supplemented with BEGM SingleQuot (Lonza).

#### McGill Laboratory – Lukacs

Human bronchial epithelia (HBE) cells *CFTR*^*F508del/F508del*^ genotype isolated from the bronchi following lung transplantation of CF individuals were gifts from Dr. W. Finkbeiner (University of California, San Francisco, CFBE13-35) or were purchased from the Cystic Fibrosis Translational Research center (CFTRc) at McGill University (BCF43 and BCF060414). HBE cells were conditionally reprogrammed according to the protocol developed by Liu and coworkers^58^. Following amplification, the cells were differentiated on Snapwell filter supports following a well established protocol^59^. Drugs were administered for 24 hours from the basolateral side mimicking systemic treatment.

#### Tigem Laboratory

Human bronchial epithelial cells obtained from a CF patient (F508del/F508del genotype) were plated on Snapwell inserts (Code 3603, Corning Life Sciences) at a density of 500,000 cells per insert, as previously described^60^. Cells were cultured for two weeks in a differentiating medium whose compositions has been previously described^60^. For the first week, the cultures were kept under liquid-liquid condition (with medium at both apical and basolateral sides of inserts). For the second week, the apical medium was removed and the epithelia were cultured in air-liquid condition (ALC).

The protocols to isolate, culture, store, and study bronchial epithelial cells from patients undergoing lung transplant were approved by the Regional Ethical Committee (Comitato Etico Regionale) under the supervision of the Italian Ministry of Health (registration number: ANTECER, 042-09/07/2018), as previously described^60^. Informed and written informed consent was obtained from all patients using a form that was also approved by the same Ethical Committee.

### Short-Circuit current measurement

#### McGill Laboratory – Hanrahan

Monolayers were incubated with vehicle (0.1% DMSO), VX-809 (1 μM) or Tα-1 (100 ng / ml) for 24 h, with cysteamine (250 μM) or cysteamine + EGCG (80 μM) for 18 h, with cysteamine for 18 h followed by 48 h washout, or with cysteamine for 18 h followed by 48 h washout in the presence of EGCG. Cells were mounted in modified Ussing chambers (Physiologic Instruments, San Diego, CA), a basolateral-to-apical NaCl chloride gradient was imposed, and transepithelial voltage was clamped at 0 mV except for 2 sec bipolar pulses to ±1 mV at 100 sec intervals to monitor resistance. Short-circuit current (Isc) across well differentiated HBE cells was measured using a VCC MC8 current-voltage clamp amplifier (Physiologic Instr.) and digitized using a PowerLab/8SP interface (ADInstruments Inc., Colorado Springs, CO) for analysis as described previously^61^. Forskolin (10 μM) was added to both sides to elicit the increase of intracellular cAMP, and this was followed by sequential additions of the potentiator genistein (50 μM) and the CFTR inhibitor CFTR_inh_-172 (10 μM) and apical ATP (100 µM) as a purinergic agonist.

#### UCSF Laboratory

Short-circuit current measurements were done as previously described^55^. FRT cells expressing F508del-CFTR were cultured on inserts (Snapwell, Corning) for 5–7 days. For corrector testing, FRT cells expressing F508del-CFTR were incubated for 18–24 hours at 37 °C with 3 μM VX-809, or 200 ng/mL human Thymosin alpha-1 peptide, or control, before measurements. The basolateral solution contained 120 mM NaCl, 3 mM KCl, 1 mM CaCl_2_, 1 mM MgCl_2_, 10 mM glucose, 25 mM NaHCO_3_, and 5 mM HEPES, pH 7.4. In the apical bathing solution, 60 mM NaCl was replaced by Na gluconate and CaCl_2_ was increased to 2 mM. The basolateral membrane was permeabilized with 250 μg/ml amphotericin B. Measurements on primary cultures of human bronchial epithelial cells were performed after 21–28 days of culture, when epithelia reached full differentiation. The primary cultures were incubated with 3 μM VX-809, or 100 ng/mL human Thymosin alpha-1 peptide, or control, at the basolateral side for 18–24 hours at 37 °C prior to measurements. The apical and basolateral chambers contained identical solutions: 120 mM NaCl, 3 mM KCl, 1 mM CaCl_2_, 1 mM MgCl_2_, 10 mM glucose, 25 mM NaHCO3, and 5 mM HEPES, pH 7.4. All solutions were bubbled with 5% CO_2_/95% O_2_ and maintained at 37 °C. Hemichambers were connected to a DVC-1000 voltage clamp (World Precision Instruments Inc., Sarasota, FL) via Ag/AgCl electrodes and 1 M KCl agar bridges for recording of the short-circuit current.

#### McGill Laboratory – Lukacs

Short-circuit current measurement of polarized HBE has been described previously^62^. Briefly, HBE were differentiated on collagen IV coated Snapwell filters under air-liquid interface for ≥4 weeks. The Snapwell filters were mounted in Ussing chambers (Physiologic Instruments) in Krebs-bicarbonate Ringer buffer (140 mM Na^+^, 120 mM Cl^−^, 5.2 mM K^+^, 25 mM HCO_3_^−^, 2.4 mM HPO_4_, 0.4 mM H_2_PO_4_, 1.2 mM Ca^2+^, 1.2 mM Mg^2+^, 5 mM glucose, pH 7.4) which was mixed by bubbling with 95% O_2_ and 5% CO_2_. Under those conditions the transepithelial resistance (TEER) was 437 ± 57 Ω*cm^2^ (mean ± S.E.M., n = 3), 419 ± 99 Ω*cm^2^ (n = 3), 455 ± 70 Ω*cm^2^ (n = 3), 495 ± 99 Ω*cm^2^ (n = 3) and 374 ± 62 Ω*cm^2^ (n = 3) for treatment with DMSO, thymosin α1, VX-809, VX-809 + thymosin α1 and cysteamine + EGCG, respectively. Measurements were performed at 37 °C in the presence of 100 μM amiloride and were recorded with the Acquire and Analyze package (Physiologic Instruments).

#### Tigem Laboratory

After two weeks of culture, epithelia were treated with vehicle (DMSO) or VX-809 (1 µM) for 24 hrs in the basolateral medium. Short-circuit current recordings were performed as previously described^60^. After treatment, Snapwell inserts were mounted in a vertical chamber resembling an Ussing system with internal fluid circulation. Both hemichambers were filled with 5 ml of a Krebs bicarbonate solution containing (in mM): 126 NaCl, 0.38 KH_2_PO_4_, 2.13 K_2_HPO_4_, 1 MgSO_4_, 1 CaCl_2_, 24 NaHCO_3_, and 10 glucose. The hemichambers were continuously bubbled with 5% CO2–95% air and the measurements were performed at 37 °C. As previously described^60^, the transepithelial voltage was short-circuited with a voltage-clamp (DVC-1000; World Precision Instruments) connected to the apical and basolateral chambers via Ag/AgCl electrodes and agar bridges (1 M KCl in 1% agar), and the offset between voltage electrodes and the fluid resistance were set to zero before experiments. The short-circuit current was recorded with a PowerLab 4/25 (ADInstruments, USA) analog-to-digital converter connected to a Macintosh computer. During recordings, epithelia were sequentially treated with: amiloride (10 µM, apical side) to block ENaC channel responsible for Na^+^ absorption; CPT-cAMP (100 µM, both apical and basolateral sides) plus VX-770 to stimulate F508del-CFTR activity; CFTRinh-172 (10 µM, apical side only) to inhibit F508del-CFTR activity. The difference between the current measured with CPT-cAMP plus potentiator and the current measured after addition of CFTRinh-172 was considered as the parameter reflecting F508del-CFTR expression in the apical membrane.

### Biochemical analysis of CFTR protein by Western Blot

Western blot analysis of endogenous CFTR protein was performed as described previously^63,64^. Briefly, whole-cell lysates of fully differentiated HBE cultures were prepared and then CFTR was immunoprecipitated. Electrophoretic separation of samples was performed on 4 to 20% gradient SDS–polyacrylamide gel electrophoresis gels (Bio-Rad) followed by transfer to a nitrocellulose membrane. Blots were probed with mouse monoclonal anti-CFTR antibodies and then with IRDye 680–goat anti-mouse immunoglobulin G (Molecular Probes). As loading control, anti-actin (Cell Signaling) was used. Protein bands were visualized using an Odyssey Infrared Imaging System (LI-COR).

### Study approval

The collection of bronchial epithelial cells and their use to investigate transepithelial ion transport were specifically approved by the Ethics Committee of the corresponding Institute following the guidelines of the corresponding national regulatory agency. Each patient provided informed consent to the study using a form that was also approved by the Ethics Committee.

### Statistics

The analysis of variance (ANOVA), followed by a post-hoc test was used in order to avoid multiple-comparisons error, since more than two groups were to be compared. The Kolmogorov-Smirnov test was used to evaluate the assumption of normality. In the case of normally distributed quantitative variables, a parametric ANOVA was performed, whereas when the quantitative variables were skewed, the nonparametric ANOVA (Kruskal-Wallis test) was applied.

Normally distributed data are expressed as the mean ± SD or mean ± SEM as indicated in the text and/or legends, and significances are 2-sided. Differences were considered statistically significant when P was less than 0.05.

## Supplementary information

Supplementary Information File

## References

[1] Castellani C, Assael BM (2017). Cystic fibrosis: a clinical view. Cell. Mol. Life Sci..

[2] Stoltz DA, Meyerholz DK, Welsh MJ (2015). Origins of cystic fibrosis lung disease. N. Engl. J. Med..

[3] Lukacs GL, Verkman AS (2012). CFTR: folding, misfolding and correcting the ΔF508 conformational defect. Trends in Molecular Medicine.

[4] Okiyoneda T, Apaja PM, Lukacs GL (2011). Protein quality control at the plasma membrane. Current Opinion in Cell Biology.

[5] Fu L (2015). ΔF508 CFTR Surface Stability Is Regulated by DAB2 and CHIP-Mediated Ubiquitination in Post-Endocytic Compartments. Plos One.

[6] Hwang T-C, Kirk KL (2013). The CFTR ion channel: gating, regulation, and anion permeation. Cold Spring Harb Perspect Med.

[7] Hanrahan JW, Sampson HM, Thomas DY (2013). Novel pharmacological strategies to treat cystic fibrosis. Trends in Pharmacological Sciences.

[8] Rowe SM, Verkman AS (2013). Cystic Fibrosis Transmembrane Regulator Correctors and Potentiators. Cold Spring Harb Perspect Med.

[9] Veit G (2016). From CFTR biology toward combinatorial pharmacotherapy: expanded classification of cystic fibrosis mutations. Mol. Biol. Cell.

[10] De Boeck K, Amaral MD (2016). Progress in therapies for cystic fibrosis. The Lancet. Respiratory Medicine.

[11] Quon BS, Rowe SM (2016). New and emerging targeted therapies for cystic fibrosis. BMJ.

[12] Zegarra-Moran O, Galietta LJV (2017). CFTR pharmacology. Cell. Mol. Life Sci..

[13] Li H, Pesce E, Sheppard DN, Singh AK, Pedemonte N (2018). Therapeutic approaches to CFTR dysfunction: From discovery to drug development. J. Cyst. Fibros..

[14] Van Goor F (2011). Correction of the F508del-CFTR protein processing defect *in vitro* by the investigational drug VX-809. Proc. Natl. Acad. Sci. USA.

[15] Wainwright Claire E., Elborn J. Stuart, Ramsey Bonnie W., Marigowda Gautham, Huang Xiaohong, Cipolli Marco, Colombo Carla, Davies Jane C., De Boeck Kris, Flume Patrick A., Konstan Michael W., McColley Susanna A., McCoy Karen, McKone Edward F., Munck Anne, Ratjen Felix, Rowe Steven M., Waltz David, Boyle Michael P. (2015). Lumacaftor–Ivacaftor in Patients with Cystic Fibrosis Homozygous for Phe508del CFTR. New England Journal of Medicine.

[16] Loo TW, Bartlett MC, Clarke DM (2013). Corrector VX-809 stabilizes the first transmembrane domain of CFTR. Biochemical Pharmacology.

[17] Ren HY (2013). VX-809 corrects folding defects in cystic fibrosis transmembrane conductance regulator protein through action on membrane-spanning domain 1. Mol. Biol. Cell.

[18] Okiyoneda T (2013). Mechanism-based corrector combination restores &Delta;F508-CFTR folding and function. Nature Chemical Biology.

[19] Hudson RP (2017). Direct Binding of the Corrector VX-809 to Human CFTR NBD1: Evidence of an Allosteric Coupling between the Binding Site and the NBD1:CL4 Interface. Molecular Pharmacology.

[20] Veit G (2018). Structure-guided combination therapy to potently improve the function of mutant CFTRs. Nat Med.

[21] Farinha CM (2013). Revertants, Low Temperature, and Correctors Reveal the Mechanism of F508del-CFTR Rescue by VX-809 and Suggest Multiple Agents for Full Correction. Chemistry &. Biology.

[22] Carlile GW (2018). A novel triple combination of pharmacological chaperones improves F508del-CFTR correction. Sci. Rep..

[23] Balch WE, Morimoto RI, Dillin A, Kelly JW (2008). Adapting Proteostasis for Disease Intervention. Science.

[24] Hutt DM (2010). Reduced histone deacetylase 7 activity restores function to misfolded CFTR in cystic fibrosis. Nature Chemical Biology.

[25] Sondo E (2018). Pharmacological Inhibition of the Ubiquitin Ligase RNF5 Rescues F508del-CFTR in Cystic Fibrosis Airway Epithelia. Cell. Chem Biol.

[26] Cheng J (2002). A Golgi-associated PDZ domain protein modulates cystic fibrosis transmembrane regulator plasma membrane expression. Journal of Biological Chemistry.

[27] Younger JM (2006). Sequential Quality-Control Checkpoints Triage Misfolded Cystic Fibrosis Transmembrane Conductance Regulator. Cell.

[28] Morito D (2008). Gp78 cooperates with RMA1 in endoplasmic reticulum-associated degradation of CFTRDeltaF508. Mol. Biol. Cell.

[29] Ye S (2010). c-Cbl facilitates endocytosis and lysosomal degradation of cystic fibrosis transmembrane conductance regulator in human airway epithelial cells. J. Biol. Chem..

[30] Okiyoneda T (2010). Peripheral protein quality control removes unfolded CFTR from the plasma membrane. Science.

[31] Tomati, V. *et al*. Genetic Inhibition Of The Ubiquitin Ligase Rnf5 Attenuates Phenotypes Associated To F508del Cystic Fibrosis Mutation. *Sci*. *Rep*, 1–17, 10.1038/srep12138 (2015). 10.1038/srep12138 PMC4505316 26183966

[32] Veit G (2016). Ribosomal Stalk Protein Silencing Partially Corrects the ΔF508-CFTR Functional Expression Defect. PLoS Biol.

[33] Tomati V (2018). High-throughput screening identifies FAU protein as a regulator of mutant cystic fibrosis transmembrane conductance regulator channel. J. Biol. Chem..

[34] Sondo E, Pesce E, Tomati V, Marini M, Pedemonte N (2017). RNF5, DAB2 and Friends: Novel Drug Targets for Cystic Fibrosis. Curr. Pharm. Des..

[35] Okiyoneda T (2018). Chaperone-Independent Peripheral Quality Control of CFTR by RFFL E3 Ligase. Developmental Cell.

[36] Luciani A (2010). Defective CFTR induces aggresome formation and lung inflammation in cystic fibrosis through ROS-mediated autophagy inhibition. Nature Cell Biology.

[37] Tosco A, De Gregorio F, Esposito S, De Stefano D, Sana I, Ferrari E, Sepe A, Salvadori L, Buonpensiero P, Di Pasqua A, Grassia R, Leone C A, Guido S, De Rosa G, Lusa S, Bona G, Stoll G, Maiuri M C, Mehta A, Kroemer G, Maiuri L, Raia V (2016). A novel treatment of cystic fibrosis acting on-target: cysteamine plus epigallocatechin gallate for the autophagy-dependent rescue of class II-mutated CFTR. Cell Death & Differentiation.

[38] Romani L (2017). Thymosin α1 represents a potential potent single-molecule-based therapy for cystic fibrosis. Nat Med.

[39] Tomati V (2018). Thymosin α-1 does not correct F508del-CFTR in cystic fibrosis airway epithelia. JCI Insight.

[40] Matthes E, Hanrahan JW, Cantin AM (2018). F508del-CFTR is not corrected by thymosin α1. Nat Med.

[41] Romani L (2018). Reply to ‘F508del-CFTR is not corrected by thymosin α1’. Nat Med.

[42] Konijnenberg A, Butterer A, Sobott F (2013). Native ion mobility-mass spectrometry and related methods in structural biology. BBA - Proteins and Proteomics.

[43] Bohrer BC, Merenbloom SI, Koeniger SL, Hilderbrand AE, Clemmer DE (2008). Biomolecule analysis by ion mobility spectrometry. Annu Rev Anal Chem (Palo Alto Calif).

[44] Ruotolo BT, Benesch JLP, Sandercock AM, Hyung S-J, Robinson CV (2008). Ion mobility-mass spectrometry analysis of large protein complexes. Nat Protoc.

[45] Elizondo-Riojas M-A, Chamow SM, Tuthill CW, Gorenstein DG, Volk DE (2011). Biochemical and Biophysical Research Communications. Biochemical And Biophysical Research Communications.

[46] Fernandez-Lima FA, Blase RC, Russell DH (2010). A Study of Ion-Neutral Collision Cross Section Values for Low Charge States of Peptides, Proteins, and Peptide/Protein Complexes. Int J Mass Spectrom.

[47] Guo Y (2015). Thymosin alpha 1 suppresses proliferation and induces apoptosis in breast cancer cells through PTEN-mediated inhibition of PI3K/Akt/mTOR signaling pathway. Apoptosis.

[48] Lao X (2015). A modified thymosin alpha 1 inhibits the growth of breast cancer both *in vitro* and *in vivo*: suppressment of cell proliferation, inducible cell apoptosis and enhancement of targeted anticancer effects. Apoptosis.

[49] Van Goor F (2006). Rescue of DeltaF508-CFTR trafficking and gating in human cystic fibrosis airway primary cultures by small molecules. AJP: Lung Cellular and Molecular Physiology.

[50] Pedemonte N (2005). Small-molecule correctors of defective DeltaF508-CFTR cellular processing identified by high-throughput screening. J. Clin. Invest..

[51] Awatade Nikhil T., Ramalho Sofia, Silva Iris A.L., Felício Verónica, Botelho Hugo M., de Poel Eyleen, Vonk Annelotte, Beekman Jeffrey M., Farinha Carlos M., Amaral Margarida D. (2019). R560S: A class II CFTR mutation that is not rescued by current modulators. Journal of Cystic Fibrosis.

[52] Davies JC (2018). VX-659-Tezacaftor-Ivacaftor in Patients with Cystic Fibrosis and One or Two Phe508del Alleles. N. Engl. J. Med..

[53] Keating D (2018). VX-445-Tezacaftor-Ivacaftor in Patients with Cystic Fibrosis and One or Two Phe508del Alleles. N. Engl. J. Med..

[54] Yamaya M, Finkbeiner WE, Chun SY, Widdicombe JH (1992). Differentiated structure and function of cultures from human tracheal epithelium. Am. J. Physiol..

[55] Haggie PM (2017). Correctors and Potentiators Rescue Function of the Truncated W1282X-Cystic Fibrosis Transmembrane Regulator (CFTR) Translation Product. J. Biol. Chem..

[56] Fulcher ML, Randell SH (2013). Human nasal and tracheo-bronchial respiratory epithelial cell culture. Methods Mol. Biol..

[57] Hild M, Jaffe AB (2016). Production of 3-D Airway Organoids From Primary Human Airway Basal Cells and Their Use in High-Throughput Screening. Curr Protoc Stem Cell Biol.

[58] Liu X (2012). ROCK inhibitor and feeder cells induce the conditional reprogramming of epithelial cells. The American Journal of Pathology.

[59] Neuberger T, Burton B, Clark H, Van Goor F (2011). Use of primary cultures of human bronchial epithelial cells isolated from cystic fibrosis patients for the pre-clinical testing of CFTR modulators. Methods Mol. Biol..

[60] Scudieri P (2012). Association of TMEM16A chloride channel overexpression with airway goblet cell metaplasia. The Journal of Physiology.

[61] Matthes E (2016). Low free drug concentration prevents inhibition of F508del CFTR functional expression by the potentiator VX-770 (ivacaftor). British Journal of Pharmacology.

[62] Veit G (2014). Some gating potentiators, including VX-770, diminish F508-CFTR functional expression. Science Translational Medicine.

[63] Cholon DM (2014). Potentiator ivacaftor abrogates pharmacological correction of F508 CFTR in cystic fibrosis. Science Translational Medicine.

[64] Gentzsch M (2016). Restoration of R117H CFTR folding and function in human airway cells through combination treatment with VX-809 and VX-770. Am. J. Physiol. Lung Cell Mol. Physiol..

