# Supplementary material for: Bioactive Thymosin Alpha-1 Does Not Influence F508del-CFTR Maturation and Activity
Source: Sci Rep. 2019 Jul 16;9:10310. doi: 10.1038/s41598-019-46639-1 (PMC6635361; doi:10.1038/s41598-019-46639-1)
Supplement: Supplementary file 1 — Supplementary Information File [file 41598_2019_46639_MOESM1_ESM.pdf]

BIOACTIVE THYMOSIN ALPHA-1 DOES NOT INFLUENCE F508del-CFTR MATURATION AND ACTIVITY

Andrea Armirotti[1], Valeria Tomati[2], Elizabeth Matthes[3], Guido Veit[3], Deborah M. Cholon[5], Puay-Wah Phuan[6], Clarissa Braccia[1], Daniela Guidone[7], Martina Gentzsch[5,8], Gergely L. Lukacs[3,4], Alan S. Verkman[6,9], Luis J.V. Galietta[7], John W. Hanrahan[3], and Nicoletta Pedemonte[2,*]

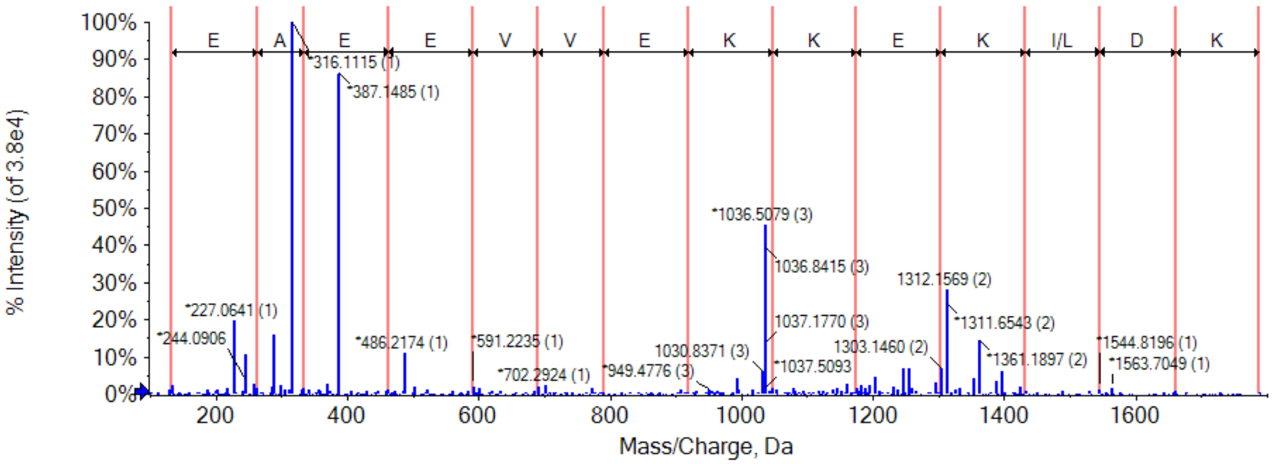

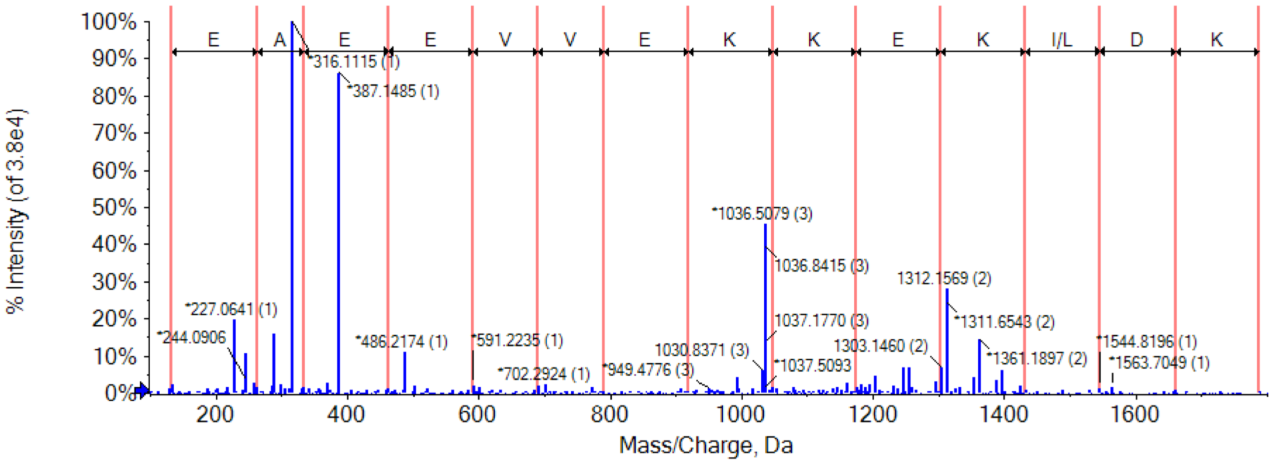

Supplementary Figure S1. High-resolution MS/MS spectra of both Abcam (top) and CRIBI (bottom) peptides, acquired using for charge state +3 (1036.5 m/z) as precursor. The plot reports a partial y-series sequencing of both peptides. The observed y- fragment ions perfectly match the **acetylated** SDAAVDTSSEITT$_{13}$KDLKEKKEVVEEAE$_{27}$N (underlined residues), indicating that in both peptides acetylation occurs before Threonine 13.

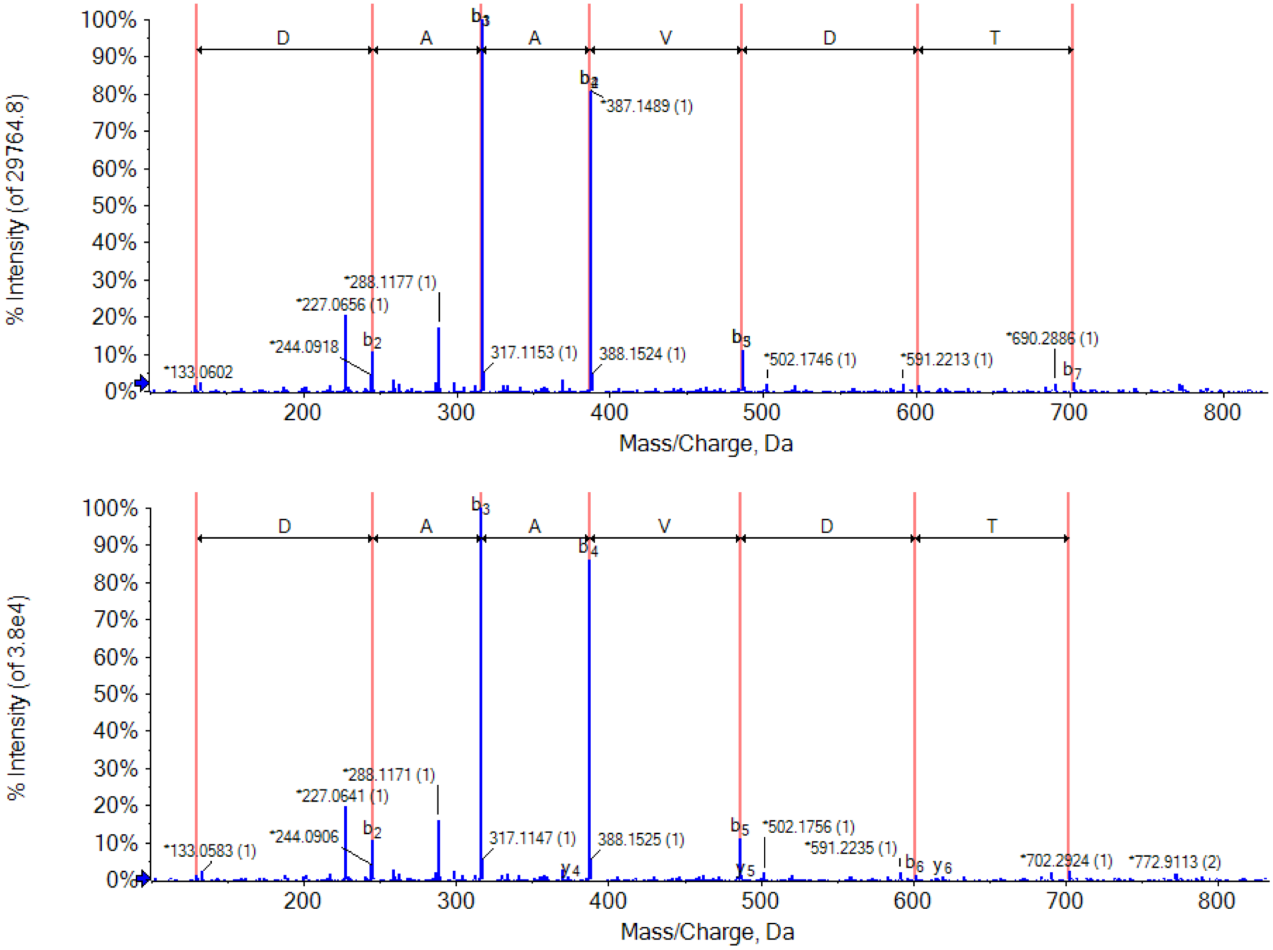

Supplementary Figure S2. High-resolution MS/MS spectra of both Abcam (top) and CRIBI (bottom) peptides, acquired using for charge state +3 (1036.5 m/z) as precursor. The plot reports a partial b-series sequencing of both peptides. The observed b- fragment ions perfectly match the **Ac-SDAAVDT**SSEITTKDLKEKKEVVEEAEN (underlined residues). These b-series fragment ions clearly indicate that the acetyl group is carried by the N-terminus of the first Serine residue.

Supplementary Figure S2

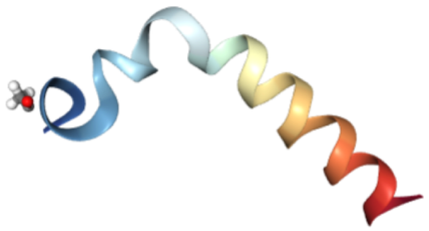

Supplementary Figure S3. NMR structure of Tα-1 with acetyl group near amino terminus (from PDB 2L9I; http://www.rcsb.org/3d-view/2L9I/0).

Supplementary Figure S3

UNCROPPED GELS FOR FIGURE 7

CFTR

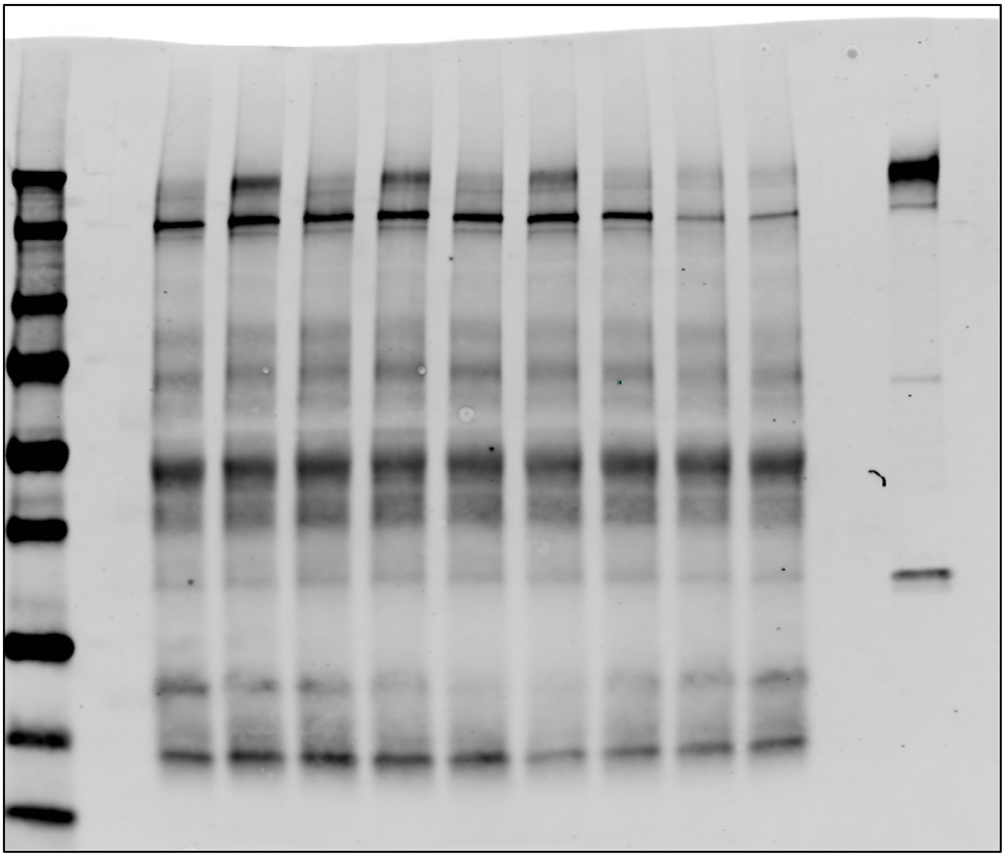

ACTIN

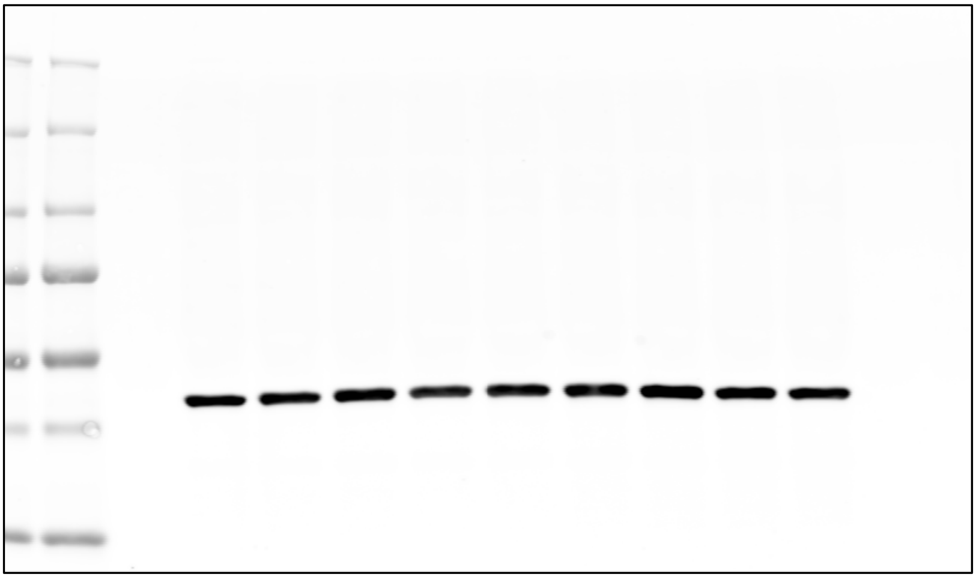

Supplementary File 1
